# Central Poststroke Pain: Mechanisms, Predictors, Clinical Assessment, and Emerging Technologies for Rehabilitation: A Structured Narrative Review

**DOI:** 10.1155/prm/2812591

**Published:** 2026-11-13

**Authors:** Cristhian Mendoza, Carolina Carmona

**Affiliations:** ^1^ Escuela de Medicina Facultad de Medicina Universidad San Sebastián Concepción 4030000 Chile uss.cl; ^2^ Department of Physical Therapy and Human Movement Sciences Feinberg School of Medicine Northwestern University Chicago Illinois 60611 USA northwestern.edu

**Keywords:** central poststroke pain, machine learning, neuropathic pain, neurorehabilitation, robotic rehabilitation

## Abstract

**Background:**

Stroke is a major cause of long‐term disability worldwide, and advances in acute care have increased the number of survivors living with chronic sequelae. Poststroke pain is a common but frequently underrecognized complication that adversely affects rehabilitation, functional recovery, psychological well‐being, and quality of life. Among its clinical forms, central poststroke pain represents one of the most challenging neuropathic pain syndromes due to its heterogeneous presentation, diagnostic complexity, and limited treatment effectiveness.

**Objective:**

This structured narrative review synthesizes current evidence on central poststroke pain, including its clinical characteristics, pathophysiological mechanisms, psychosocial impact, risk factors, and therapeutic approaches. It also examines the emerging role of machine learning and robotic rehabilitation while proposing a clinical diagnostic framework and an evidence‐informed therapeutic hierarchy for central poststroke pain management.

**Methods:**

A narrative literature review informed by a structured literature search was conducted using PubMed and Web of Science databases through November 2025. Clinical studies, reviews, systematic reviews, clinical guidelines, and expert consensus documents relevant to central poststroke pain were identified and synthesized thematically.

**Results:**

Central poststroke pain remains underdiagnosed and undertreated, largely due to its clinical heterogeneity and the absence of standardized diagnostic pathways. Current evidence supports a multimodal approach that combines pharmacological, neuromodulatory, and rehabilitative interventions, with duloxetine, amitriptyline, and repetitive transcranial magnetic stimulation demonstrating the strongest evidence for pain relief. Emerging technologies such as machine learning and robotic rehabilitation show potential for improving assessment and personalization of care; however, central poststroke pain‐specific predictive models remain unavailable and evidence supporting pain‐related outcomes remains limited.

**Conclusion:**

Central poststroke pain continues to represent an important unmet need in stroke rehabilitation. Future advances will likely depend on improved diagnostic frameworks, personalized multimodal management strategies, and rigorous validation of emerging technologies before their widespread clinical implementation.

## 1. Introduction

Stroke (cerebrovascular accident, CVA) remains, among chronic noncommunicable diseases, the second leading cause of mortality and the third leading cause of combined mortality and disability worldwide [1]. Timely access to healthcare systems and technological advances in emergency care have contributed to a growing population of stroke survivors [2]. These individuals frequently experience numerous secondary complications, including physical disability, reduced mobility [3], cognitive impairment, depression, and chronic pain [4–7].

Poststroke pain (PSP) has an estimated prevalence ranging from 11% to 66% [8]. This condition is a critical factor for discontinuation of rehabilitation services and significantly reduces quality of life, independence, overall functioning, and mental health. PSP is also associated with increased risk of recurrent stroke and higher mortality rates [2, 9]. Despite its profound impact, PSP remains largely overlooked in diagnostic, assessment, and treatment programs for this population.

Among PSP manifestations, central poststroke pain (CPSP) represents one of the most complex and challenging conditions to manage. CPSP is a chronic neuropathic pain syndrome that arises as a direct consequence of injury or dysfunction within the central nervous system (CNS) caused by stroke [10].

Moreover, while many poststroke complications have been widely studied, CPSP remains underdiagnosed and undertreated. This is partly because its clinical presentation varies and there are no widely accepted standardized diagnostic tools.

CPSP exhibits considerable heterogeneity in onset (early or delayed), anatomical distribution, intensity, and symptom progression, complicating timely diagnosis and treatment. Consequently, CPSP is frequently underdiagnosed and undertreated, becoming a significant contributor to physical and emotional deterioration in stroke survivors. This, in turn, adversely affects functional recovery, morbidity, mortality, and overall quality of life.

In this context, distinguishing CPSP from other types of PSP is essential because its central neuropathic origin involves distinct pathophysiological mechanisms and therapeutic responses. As a result, conventional treatments for nociceptive or peripheral pain are often less effective for CPSP.

Several studies report that up to one‐third of individuals poststroke develop CPSP, with some prevalence estimates exceeding 40% [7], underscoring the magnitude of this condition and the challenge of early detection, establishing appropriate evaluation and assessment tools, and management protocols.

Emerging technologies driven by robotic and digital health transformation [11] are redefining approaches to complex neurological conditions. These innovations offer advanced tools that integrate big data analytics, imaging [12], clinical process automation, and optimization of functional rehabilitation.

In this context, artificial intelligence (AI) and machine learning (ML), combined with robotic rehabilitation systems, demonstrate significant potential to complement traditional methods [11, 13]. They enable more objective assessment of motor performance, dynamic personalization of therapeutic interventions, and more accurate prediction of clinical outcomes.

However, while these technologies have advanced significantly in poststroke motor rehabilitation, their use in managing central pain remains in its early stages and requires a thorough, critical evaluation of the current evidence.

Such advancements contribute to the development of clinical sciences that are preventive, accurate, and patient‐centered.

This review aims to synthesize current evidence on CPSP by examining its functional, emotional, and psychosocial implications; identifying key risk factors and predictors for its development; and evaluating both pharmacological and nonpharmacological therapeutic strategies, including neuromodulation and robotic rehabilitation technologies. Furthermore, it explores the emerging role of ML as a transformative tool for early detection, functional prognosis, and personalized interventions within this population.

## 2. Methods

A narrative literature review informed by a structured literature search to synthesize current evidence on CPSP. The review focused on CPSP’s clinical characteristics, current rehabilitation strategies, and technology‐assisted interventions.

The literature search was conducted in the PubMed and Web of Science databases through November 2025. The search strategy combined Medical Subject Headings (MeSH) and free‐text terms related to PSP and neurorehabilitation. The terms used included “poststroke central pain,” “central neuropathic pain,” “chronic pain after stroke,” “neuropathic mechanisms,” “rehabilitation strategies,” “nonpharmacological interventions,” “robotic rehabilitation,” “neuromodulation,” “transcranial magnetic stimulation,” and “technology‐assisted rehabilitation.” Boolean operators (AND, OR) were used to refine and optimize the search results. No language restrictions were applied.

Articles were selected for their relevance to the review’s objectives, with priority given to original clinical studies, experimental research, narrative and systematic reviews, clinical practice guidelines, and expert consensus documents. Particular attention was paid to studies on emerging rehabilitation technologies, such as robotic systems, neuromodulation techniques, and AI‐based approaches.

The selection process followed a structured approach, in which titles, abstracts, and full texts were reviewed to identify the most relevant evidence. This procedure was not intended to meet the methodological criteria of a formal systematic review, but rather to ensure broad, representative, and relevant and meaningful coverage of the available literature.

Exclusion criteria comprised duplicate publications, articles without full‐text access, studies not directly related to CPSP or stroke rehabilitation, and non‐peer‐reviewed literature, including editorials, commentaries, and conference abstracts.

Information was synthesized qualitatively and organized thematically to provide an integrative overview of current knowledge, identify gaps in the literature, and highlight future directions for CPSP treatment and rehabilitation.

This approach allows for the integration of heterogeneous evidence while maintaining methodological transparency, which is particularly relevant in areas such as CPSP, where the available evidence is diverse, inconsistent, and still lacks methodological standardization.

Given the narrative nature of this review, a formal PRISMA flow diagram was not developed. Nevertheless, the search strategy was structured and systematic to ensure broad and representative coverage of the available literature on CPSP.

## 3. Results and Discussion

### 3.1. Stroke (CVA)

Stroke remains a leading cause of disability and mortality worldwide [14], resulting in numerous long‐term sequelae that significantly affect individuals’ functionality and quality of life [15]. Among these complications, PSP [16] poses a significant clinical challenge during rehabilitation. CPSP, in particular, is notable for its neuropathic origin, heterogeneous clinical presentation, and substantial impact on functional recovery, making it a complex and often underdiagnosed condition.

### 3.2. Acute and Chronic Pain

The International Association for the Study of Pain (IASP) defines pain as “an unpleasant sensory and emotional experience associated with, or resembling that associated with, actual or potential tissue damage” [17]. Pain is a major global public health issue, affecting approximately 20% of the population worldwide and more than 1.5 billion people living with chronic pain [18, 19]. Prevalence increases with age, reaching up to 50% among community‐dwelling older adults [20, 21]. In Chile, a recent cross‐sectional study reported an overall prevalence of chronic pain of 28.9% among individuals over 15 years old, increasing to 40.2% in those aged 65–80 years [22].

Pain can be classified according to duration and mechanism. Acute pain is generally a protective response, whereas chronic pain is associated with neuroplastic, inflammatory, and neurotransmission changes that contribute to its persistence [23].

Three pain types are recognized: nociceptive, neuropathic, and nociplastic [24]. Neuropathic pain has received particular attention because of its prevalence, complexity, and impact on quality of life [25]. It affects 7%–10% of the general population and represents 20%–25% of chronic pain cases [26]. Alterations in somatosensory pathways following central or peripheral nervous system injury play a key role in its development and chronification [27].

According to the IASP, chronic pain persists beyond normal tissue healing and may continue for months or years, even in the absence of an identifiable cause [28]. Its prevalence ranges from 11% to 40% in adults [29], and is associated with significant effects on physical, emotional, and social well‐being [30, 31]. It is important to note that chronic pain accounts for 71% of global disability (Global Burden of Disease Study 2016) [32]. Various studies have shown that chronic pain increases with age, is reported more frequently by women than men, and that pain intensity and related disabilities are more common in women [29].

Chronic pain is among the leading causes of years lived with disability and generates substantial social and economic burden [33]. Its multidimensional nature complicates treatment, and nearly two‐thirds of patients report dissatisfaction with current management [34]. Consequently, comprehensive assessment remains a key component of pain care [35].

Chronic pain restricts physical activity, daily functioning, social participation, and work performance while contributing to emotional distress, mood disturbances, and financial burden [36, 37]. In a study of 833 patients with chronic pain, more than 50% reported personality changes, anxiety, depression, and/or sleep disturbances [36]. In specific cases such as fibromyalgia or nonspecific low back pain, chronic pain has come to be considered a disease in its own right [38] and is even identified as a significant risk factor for suicide [39, 40].

Accurate pain assessment is essential for effective management, although inadequate evaluation remains one of the main barriers in clinical practice [41]. In stroke rehabilitation, clinicians should recognize pain as a frequent complication, identify individuals at higher risk, and systematically assess its presence and characteristics [8].

### 3.3. PSP

Stroke causes physical, psycho‐emotional, behavioral, and communication changes that transform the lives not only of patients but also of their caregivers. Although several previous clinical studies have analyzed the impact of age, gender, cognitive status, depression, and comorbidities on poststroke rehabilitation [42], pain (a critical factor) has been underestimated despite its documented impact on functionality, quality of life, and adherence to rehabilitation.

PSP is defined as any pain occurring after a stroke diagnosis, excluding pre‐existing conditions such as cancer‐related or diabetic pain. Initially, various studies attributed PSP solely to thalamic involvement (mainly the ventral posteromedial, posterolateral nuclei, and possibly the pulvinar nucleus). Currently, lesions at any level of the spinothalamic tract (including the lateral medulla, pons, lenticular‐capsular region, and cortex) are also considered potential contributors to PSP development [13]. Evidence from other neurological and traumatic conditions suggests that early alterations in brain connectivity may increase vulnerability to the later development of chronic pain [43].

Recent studies indicate that close to 60% of individuals with stroke experience pain, and nearly 40% continue to experience pain 5 years post‐onset, significantly impacting quality of life and independence in activities of daily living [44]. PSP has also been associated with higher poststroke mortality risk [45, 46] and dependency [47].

Current evidence, with highly variable estimates, shows PSP prevalence ranging from 14% to 70% [48] and between 11% and 48% 1 year after stroke [49]. In a cross‐sectional study by Westerlind et al., with 5‐year follow‐up, PSP was estimated in 10%–50% of individuals with stroke [2], of whom 70% reported daily pain [8].

The following types of PSP are currently recognized: central neuropathic pain, complex regional pain syndrome (CRPS), pain associated with spasticity, poststroke headache, and shoulder pain [50].

Untreated or inadequately managed PSP is strongly associated with poorer functional outcomes, increased rehabilitation dropout, higher risk of recurrent stroke, and elevated mortality rates [2, 9, 47, 51, 52]. Furthermore, PSP has been linked to suicidal ideation [53]. Edwards et al. emphasize that effective management begins with accurate recognition and assessment [54]. However, PSP remains underestimated and poorly addressed, leading to neglected or inefficient pain evaluation. This reflects a limited understanding of its underlying mechanisms and contributes to inadequate clinical management [48].

In this context, CPSP, characterized as neuropathic, is the least recognized, studied, and considered—even ignored—in the evaluation, follow‐up, and treatment of stroke survivors, which greatly impacts recovery [55]. Due to the lack of a standardized or gold standard pain assessment for individuals poststroke and the wide variety of available tools, healthcare professionals face challenges in determining the most appropriate evaluation approach for their patients [54].

### 3.4. CPSP

CPSP is defined as pain originating from an injury to the CNS [55, 56] and occurring because of cerebrovascular diseases, such as stroke [57], including lesions in the brainstem, thalamus, and cerebral cortex [10]. This pain may manifest in one side of the body, appear in multiple areas, or be limited to smaller regions [58]. Overall, it is estimated that one‐third of poststroke cases develop CPSP [59]. However, this condition remains underdiagnosed due to its complex clinical presentation and frequent overlap with other types of PSP. In this context, the observations of Djojoseputro et al. and other authors are relevant, emphasizing the need for reliable biomarkers or reference diagnostic tools for better identification of this condition [44].

From a clinical perspective, CPSP should be understood as severe chronic neuropathic pain, often refractory to conventional treatments, directly impacting patients’ psychosocial and economic well‐being and reducing quality of life, while also being associated with low therapeutic satisfaction [60, 61]. This complexity makes CPSP management a major challenge for healthcare professionals, where current technological tools and the use of AI emerge as potentially valuable resources for rehabilitation teams and patients alike.

It is important to note that neuropathic pain was defined by the IASP in 1994 as “pain initiated or caused by a primary lesion or dysfunction in the nervous system,” observed across a wide range of syndromic diagnoses and characterized by a discrepancy between objective signs of tissue damage, its clinical magnitude, and associated disability. These differences often lead to underdiagnosis and complicate therapeutic approaches. Currently, to improve understanding and management of this condition, the IASP redefines neuropathic pain as “pain caused as a direct consequence of a lesion or disease affecting the somatosensory system” [44, 62]. According to Hayes’s 2007 study, among patients attending pain clinics reporting chronic neuropathic pain, 78% continued to experience it after 6 months, decreasing to 56% after 12 months [63].

Recent studies show that CPSP can develop in any CNS location affected by stroke that impacts pain pathways and processing regions. Thus, any lesion along the ascending somatosensory pathways can trigger CPSP [44]. Clinically, its presentation does not differ significantly from other neuropathic pain disorders [64].

Nevertheless, evidence regarding the diagnosis and assessment of central neuropathic pain in neurorehabilitation is still limited. This is partly due to differences in selection criteria and the absence of biomarkers to distinguish it from other concomitant pain syndromes [65, 66], including those arising from conditions outside the CNS [67].

CPSP is one of the most significant chronic physical and mental symptoms in stroke survivors, interfering substantially with daily activities, reducing physical tolerance, and diminishing rehabilitation effectiveness, thereby increasing the caregiving burden on families. In this regard, van Driel et al., in a prospective study on patients undergoing various surgical procedures, designed and validated two predictive models aimed at early identification of those at risk of developing chronic postoperative pain, to alert medical staff for appropriate evaluation and treatment [68]. Consequently, early prediction of central pain in poststroke individuals becomes crucial.

Fillingim and collaborators applied ML techniques to multidimensional biological modalities to derive candidate biomarkers for chronic pain using data from the UK Biobank. Their results showed that pain prediction can be achieved accurately by combining biological and psychosocial factors. However, they observed that reported pain predominantly depends on psychosocial factors, questioning the existence of a single biological marker for predicting subjective chronic pain and suggesting that future studies should prioritize integrating these psychosocial factors [69].

The incidence of CPSP remains uncertain, with highly variable estimates. For context, a European systematic review reported that neuropathic pain affects between 6.9% and 10% of the general population [70]. This variability is attributed to etiological heterogeneity, nonspecific lesion location, study design, and timing of patient evaluation after stroke [71]. For example, MacGowan et al., in 1997, observed that among 63 patients with bulbar infarction, 25% developed CPSP, using tools such as quantitative sensory testing (QST) [72].

In summary, biomarkers have the potential to greatly improve the diagnosis, prognosis, and treatment of chronic pain across different comorbidities. However, their use in clinical practice is still limited because of the complex and variable nature of chronic pain. Developing composite biomarkers appears to be more effective than relying on single diagnostic tests. While advanced techniques like MRI and EEG can provide valuable information, they are not practical for routine use. Still, algorithmic analyses of these data sources have shown promising links with pain intensity [73].

To date, the largest CPSP prevalence study, conducted in 695 health centers across 35 countries with a cohort of 15,754 eligible individuals, found that 10.6% of participants (1665 individuals) developed chronic PSP, of whom 431 (25%) were diagnosed with CPSP [51]. More recent studies reinforce this trend, indicating that at least one‐third of poststroke cases present CPSP [59, 74]. Similarly, a cross‐sectional study in Indonesia reported a prevalence of 30.1% of CPSP in stroke patients [75], confirming that it is a frequent, persistent, and clinically significant condition.

This variability may be explained by differences in diagnostic criteria, timing of assessment after stroke, lesion characteristics, and heterogeneity in study populations, highlighting the complexity of accurately estimating CPSP prevalence.

In this scenario, it is essential to implement early detection strategies, standardize diagnostic criteria, and develop innovative therapeutic plans to improve the quality of life of individuals facing this complex poststroke condition.

#### 3.4.1. Pathophysiology

The transition from acute to chronic pain is a complex process involving changes in both the CNS and peripheral tissues. These changes include alterations in ion channels, inflammatory mediators, synaptic plasticity, and neuroinflammation, as well as genetic, epigenetic, and psychosocial influences [76].

Numerous studies have shown that chronic pain induces neuroplastic changes, leading to decreased gray matter volume and altered functional connectivity in brain and subcortical regions responsible for processing pain. These regions include the anterior cingulate cortex, insula, prefrontal cortex, thalamus, and hippocampus. Such changes affect both the sensory and emotional aspects of pain, contributing to its persistence through maladaptive neural circuits and negative feedback loops [28].

Despite its impact on quality of life, CPSP is an under‐recognized complication of stroke, and its pathophysiology has not been fully elucidated. Multiple factors have been proposed to contribute to CPSP, including sensory deafferentation, spinothalamic dysfunction, and central sensitization and disinhibition in pain networks [77]. As noted by Betancur et al., CPSP is possibly due to the loss of somatosensory integration, along with alterations in functional and cortical plasticity [13].

Additionally, although there is evidence linking CPSP to spinothalamic lesions, the literature reports inconsistent findings. Lesion location alone does not fully explain the clinical variability observed in CPSP. This suggests that CPSP cannot be understood by structural factors alone; it also requires consideration of changes at the neuronal level and dynamic sensory integration processes.

Two recent theories explain the development of CPSP. The first focuses on lesions along the ascending somatosensory pathways that generate pain; the second theory explores the imbalance between excitatory and inhibitory inputs in the systems involved. Both mechanisms can occur simultaneously [44, 62, 78].

From this perspective, pathophysiological models should be viewed as complementary rather than mutually exclusive. The coexistence of mechanisms, such as central sensitization, disinhibition, and excitatory/inhibitory imbalance, reinforces the multifactorial nature of CPSP and highlights the difficulty of explaining it through a single hypothesis.

Other articles emphasize that alterations in neuronal plasticity seem to be the primary mechanism underlying the development of CPSP, supported by similar pathophysiological findings in other neuropathic pain disorders; in this context, abnormal spontaneous pain is associated with inadequate responses driven by hyperexcitability in the cortex and thalamus [72].

The pathophysiological processes of CPSP involve complex interactions among neurochemical, excitotoxic, and inflammatory mechanisms, along with central sensitization, neuronal hyperexcitability, dysfunction of the descending pain modulatory system, loss of GABAergic interneurons, microglial activation, and morphological alterations. Several authors have proposed that these mechanisms can be interpreted mainly under three theories: central disinhibition, central imbalance, and central sensitization [10]. More recent research suggests that central sensitization and central neurological processes may influence overall pain perception [79].

This group of mechanisms explains not only the persistence of pain but also the variability in therapeutic responses observed among patients. These differences contribute to inconsistent results in clinical studies and highlight the need for individualized therapeutic approaches.

We must highlight that stroke induces a pro‐inflammatory glial phenotype in the injured tissue, leading to significant production of cytokines and inflammatory mediators that, at the same time, generate central sensitization, which is directly related to the final appearance of CPSP.

#### 3.4.2. General Clinical Features

The clinical features of CPSP stem from its underlying pathophysiological mechanisms. CPSP typically presents with a wide range of symptoms, which are often heterogeneous and nonspecific.

Central pain can develop as early as 1 month after a stroke or as late as one to 6 years afterward [65, 80]. In about 25% of cases, CPSP appears during the acute or subacute phase following a stroke; however, it most commonly develops between three and 6 months after the injury [81]. Both early and late onsets have been reported [10, 81]. For example, Bashir et al. found that 50% of subjects experienced pain 3 months after their stroke [80].

The clinical characteristics of CPSP resemble those of other central and peripheral neuropathic pain conditions, and they lack distinctive features or consistent signs in terms of onset, presentation, and intensity [82].

Pain symptoms in patients with CPSP show considerable variability but commonly include dysesthesia, paresthesia, allodynia (to touch, cold, or movement), and hyperalgesia [83–85]. These sensory phenomena have been reported in up to 85% of patients with CPSP [10, 86]. More than 90% of patients experience abnormalities in thermal sensations, particularly cold, while sensory deficits in other modalities, such as touch and vibration, are less common [82].

The pain is moderate to severe, usually rated 3 to 8 on the visual analog scale (VAS), and is diffuse, encompassing extensive somatic areas. Generally, the painful area corresponds to the affected brain structures, but this correlation is not found in all cases [87].

#### 3.4.3. Diagnostic Criteria

Many neurorehabilitation societies recommend the diagnostic criteria for CPSP by Klit et al. [10]. Its primary criteria indicate pain in an area of the body corresponding to the CNS injury, history of previous stroke and onset of pain after or during the ictal episode, confirmation of CNS injury by imaging or negative or positive sensory signs circumscribed to the area of the body corresponding to the injury, other excluded or unlikely causes. While the criteria that support the diagnosis are the absence of a relationship with movement, inflammation, or damage to the local tissue in the area of pain, burning, cold, electric, stabbing, or oppressive quality; although any description is valid, allodynia or dysesthesia to the touch or cold.

On the other hand, the diagnostic criteria for chronic CPSP of the ACTTION American Pain Society Pain Taxonomy (AAPT), adapted from Klit et al. [10, 62], in 2017, consider a diagnostic test that confirms a stroke or a history strongly suggestive of stroke (defined as a diagnostic test that confirms a stroke or a history strongly suggestive of stroke (defined as a neurological deficit of cerebrovascular origin that persists for more than 24 h); continuous or recurrent pain after a stroke, with pain onset at the time of the stroke or up to 1 year after stroke onset and a pain duration of at least 3 months; the pain is described in the body area affected by the stroke and is associated with sensory changes in the same neuroanatomically plausible distribution, as indicated by the presence of at least one positive sensory sign (e.g., dynamic mechanical allodynia or response to cold) or one negative sensory sign (e.g., elevated cold or heat thresholds or decreased sensitivity to touch, pinprick, or thermal stimuli); and finally, that there is no other diagnosis that better explains the pain [58].

Notably, even with the use of screening tools, 10%–20% of patients with neuropathic pain are not correctly identified. This underscores the importance of thorough clinical evaluation [88]. Another important issue is that some people are unable to self‐report pain after a stroke, making it challenging to accurately estimate how common this condition truly is [89]. Additionally, because sensory abnormalities often occur over large areas of the body in these patients (whether or not they have pain), a sensory examination alone cannot definitively determine if the pain is neuropathic.

Currently, there is no evidence‐based classification system that provides a clear and unified diagnosis for central neuropathic pain in people after stroke. Such a system would need to standardize and connect the various contributing factors (physical, psychological, and social) while also distinguishing this condition from other painful comorbidities. This lack of standardization was highlighted by Widerström‐Noga et al. in 2017 [58].

Therefore, understanding the clinical variables, modifiable factors, and consequences of CPSP is essential for designing specific and effective interventions for people with PPPC [46].

#### 3.4.4. Functional, Emotional, and Psychosocial Impact

CPSP has a psychological impact on patients, as it reduces sleep quality and interferes with basic activities of daily living (BADL), hindering rehabilitation efforts [71]. We must consider that sleep is vital for the functioning of our organic, endocrine, and neurological systems, and fundamental for memory and learning processes, for the conservation of energy, and brain plasticity.

Persistent pain in the CPSP affects psychological well‐being by altering normal sleep‐wake patterns, as well as reducing social interaction; in addition, chronic pain itself produces cognitive impairment and depression in this group of people [85]. Approximately one in two individuals post‐suffer from anxiety and depression during the first year after stroke [90]. Among patients who developed depression within 3 months poststroke, approximately half remained depressed at 1 year [91].

On the other hand, a lack of sleep translates, for example, into alterations in the perception of pain, overall quality of life, and lower life expectancy [92, 93]. As a factor to be highlighted, it has also been reported that about 50%–60% of people poststroke report symptoms associated with insomnia [94, 95], where the greatest contributor to this process is pain, ultimately influencing the recovery process [96].

CPSP can seriously condition a person’s ability to perform their ADLs, alter their mood and sleep, trigger psychiatric disturbances with suicidal thoughts (people with stroke accompanied by CPSP have a risk of suicide three times higher than in the absence of CPSP), and markedly decrease quality of life [86].

#### 3.4.5. Risk Factors and Predictors

Although specific evidence on predictors in CPSP remains limited, findings from CPSP can serve as a valid reference, as they share multiple pathophysiological mechanisms and risk factors.

Predictors such as female sex, cognitive impairment, increased stroke severity, functional dependence, fatigue, excessive alcohol consumption, diabetes mellitus, depression, antithrombotic treatment, and statin use have previously been associated with an increased likelihood of developing PSP [2, 51]. Regardless of their etiology, patients with neuropathic pain typically report significantly higher pain intensity compared to those without this condition, with average values of 6.4 versus 4.6 on a scale of 0–10 [97, 98].

Additionally, Mohanan et al. highlight that younger age, the specific location of brain injury, and smoking could be additional factors associated with an increased risk of developing CPSP [65].

#### 3.4.6. Treatment and Therapeutic Challenges

Overall, it is estimated that approximately 25% of patients with brain injury experience pain, negatively affecting their quality of life and adherence to neurorehabilitation programs [99].

Although various medications and neuromodulatory approaches have demonstrated efficacy in relieving peripheral neuropathic pain, their impact on CPSP has been limited [100, 101]. A recent systematic review underscores the urgent need for additional studies to define more specific treatment and rehabilitation interventions for this type of pain, emphasizing the importance of differential evaluations that enable adequate clinical characterization [33].

The Fugl‐Meyer assessment (FMA) [102–104], widely recognized for its high reliability and validity, is considered the gold standard for evaluating poststroke sensorimotor function [105, 106]; however, the pain sensitivity item addresses only nociceptive peripheral pain and does not capture manifestations of chronic neuropathic pain. This limitation underscores the need for specific tools to assess central CPSP, as this condition can significantly hinder rehabilitation. Therefore, a comprehensive understanding of CPSP (including its etiology, pathophysiology, clinical features, and assessment methods) is essential to optimize treatment and improve functional outcomes in poststroke patients [107].

#### 3.4.7. General Management of the CPSP

Due to the heterogeneity of underlying factors, variability in clinical symptoms, and the complexity of rehabilitation, CPSP requires not only early and accurate differentiation from other PSP syndromes but also timely, effective, patient‐centered, and multifactorial treatment to improve quality of life. Pain management during neurorehabilitation is challenging and demands an appropriate combination of pharmacological and nonpharmacological strategies. Therefore, thorough assessment and accurate diagnosis of pain manifestations are essential to recognize, address, or prevent pain during rehabilitation after brain injury [86]. As noted by several authors, “effective management of chronic poststroke pain depends on recognizing and distinguishing it from coexisting poststroke pain” [65]. Overall, adequate treatment of CPSP not only alleviates pain but also supports rehabilitation and functional recovery [108].

#### 3.4.8. Pharmacological Treatment

Regarding neuropathic pain, practice guidelines such as those from the Italian Consensus Conference on Pain in Neurorehabilitation (ICCPN) recommend the following pharmacological treatments: (A) High‐first‐line options include tricyclic antidepressants, selective serotonin reuptake inhibitors (SSRIs), pregabalin, and gabapentin; (B) second‐line therapies are 5% lidocaine patches, 8% capsaicin patches, and tramadol; (C) third‐line options are strong opioids and botulinum toxin. Studies focused on the treatment of CPSP are scarce, and most rely on pharmacotherapy, with only a minority of patients responding [86].

Some clinical trials have shown a reduction in pain in CPSP with acupuncture and apipuncture, but with the limitations of an effect only perceptible for a short period and in small population samples [85, 109, 110].

In a systematic review conducted by Mulla et al. in 2015, based on eight eligible randomized clinical trials with a total of 459 patients, in which pharmacological and nonpharmacological therapies were performed in CPSP, the authors conclude that their findings are inconsistent with the main clinical practice guidelines and that the available evidence, at that time, it does not suggest benefits of any therapy presented in clinical trials [110]. It is important to note that, in these studies, the evaluation and diagnostic processes are highly diverse and, in many cases, not clearly described or well defined.

After thoroughly reviewing the literature, Asadauskas et al. found that pharmacological treatment for CPSP primarily relies on antidepressants, anticonvulsants, and opioid analgesics. While antidepressants and anticonvulsants are generally recommended as first‐line treatments, the authors note significant variability in how patients respond to these medications. They emphasize that current research limitations make it clear that a more personalized, multimodal treatment approach is needed [82]. The study also highlights that, among nonpharmacological treatments, noninvasive brain stimulation (specifically repetitive transcranial magnetic stimulation, rTMS) and physical therapy appear to be the most effective options for managing CPSP.

Liampas et al., in their study on the prevalence and management of CPSP, conclude that future research should focus on well‐designed, randomized clinical trials of pharmacological and nonpharmacological interventions aimed at alleviating CPSP, a painful syndrome that is more common than we think and often totally neglected [6].

#### 3.4.9. Noninvasive Neuromodulation

In a recent systematic review and meta‐analysis, conducted by Tamasauskas et al. (2025), which included 42 original studies, aimed to understand the efficacy of available pharmacological, physical, psychological, and neuromodulation interventions to reduce pain in patients with CPSP, it is highlighted that duloxetine, amitriptyline, and rTMS presented the strongest evidence of their efficacy in relieving pain induced in this condition. In addition, the need for methodologically adequate randomized clinical trials with larger population samples to determine the effectiveness of acupuncture and virtual reality, as well as invasive and noninvasive neuromodulation treatments, in this condition is argued [7]. rTMS is increasingly being explored for the management of neuropathic pain in general and has been widely recommended for patients who are refractory to pharmacological therapy [111]. In general, rTMS is beneficial for temporary pain relief in cases such as CPSP; however, more studies are required to determine its long‐term effects on pain relief, especially in chronic pain conditions [72].

To assess the efficacy and quality of studies on noninvasive brain stimulation (tDCS and rTMS) for CPSP, Ramger et al. conducted a systematic review in 2019. Six studies met the eligibility criteria: four controlled trials (one randomized and three nonrandomized) and two descriptive studies (case series), comprising 109 participants. The authors concluded that noninvasive motor cortex stimulation may have a therapeutic effect on pain levels in individuals with CPSP, as evidenced by significant reductions in pain intensity and pain threshold scores [112].

According to Yang et al., there is currently no standardized treatment protocol for rTMS in CPSP, as variations in stimulation parameters (such as frequency, site, and duration) yield inconsistent analgesic outcomes [113].

In a retrospective cohort study analyzing 12 years of clinical data to evaluate the long‐term analgesic effects of rTMS on the primary motor cortex in patients with poststroke neuropathic pain, between 25% and 59% of those with drug‐refractory neuropathic pain experienced clinical benefits from repeated rTMS sessions, with some achieving prolonged pain relief lasting several years. Success rates were higher among patients with central PSP compared to peripheral PSP [114].

A systematic review by Gurdiel‐Álvarez et al. aimed to evaluate the efficacy of rTMS in CPSP patients. Nine studies were included, six of which (four RCTs and two non‐RCTs) were incorporated into a meta‐analysis. Results indicated that, although the available evidence is of low quality, the effect size of rTMS in reducing CPSP intensity was considerable [115].

Consistent with these findings, Xu et al. conducted a systematic review to assess the efficacy and safety of nonpharmacological therapies for CPSP. This review included 11 controlled clinical trials with 166 participants. The authors concluded that while these interventions (invasive electrical brain stimulation, repetitive transcranial magnetic stimulation, transcranial direct current stimulation, caloric vestibular stimulation, and acupuncture) show promising preliminary results, the evidence remains insufficient and requires confirmation through rigorous randomized clinical trials. They also highlighted the poor methodological quality of the included studies, noting that only one presented a low risk of bias [116].

These inconsistencies likely stem from differences in stimulation parameters, study designs, and patient selection. Such variability limits the generalizability of findings and underscores the need for standardized protocols. Due to these limitations and the challenges of conventional approaches, emerging technologies are being explored as promising tools to improve the assessment and rehabilitation of individuals with CPSP.

#### 3.4.10. Emerging Technologies: ML and Robotic Rehabilitation

Emerging technologies may contribute to advances in the assessment and management of CPSP, although current evidence remains limited. Importantly, ML and robotic rehabilitation represent distinct approaches, and potentially complementary strategies.

##### 3.4.10.1. ML

ML refers to computational methods that identify patterns within large datasets to develop predictive or classification models that can support diagnosis, risk prediction, and personalized clinical decision‐making [117]. In stroke, ML has been applied to predict outcomes such as cognitive decline, fatigue, mortality, and functional recovery, demonstrating potential utility for risk stratification, diagnosis, assessment, prognosis, functional recovery, and disability, and its potential to support individualized care and outcome prediction [118–122]. Kokkotis et al. [120] highlighted the potential of ML approaches to predict functional outcomes after stroke by incorporating multidisciplinary data sources, such as clinical and patient‐related characteristics. While ML may offer opportunities to improve outcome prediction and rehabilitation planning, their added value over conventional prediction methods and their generalizability require further investigation. Similarly, Radley et al. [121], in a systematic review of ML models for predicting poststroke mortality, found a high overall risk of bias across all included studies, raising concerns about model reliability and readiness for clinical implementation. These findings underscore the need for rigorous methodological approaches, independent external validation, and evaluation of clinical utility before ML‐based prediction models can be routinely integrated into stroke care.

Despite these advances, no ML models have been specifically developed and validated for CPSP, representing a critical opportunity for clinical and translational research.

In pain research, ML algorithms have identified individualized neural activity patterns associated with chronic pain syndromes and have been used to investigate candidate pain biomarkers [111, 123]. Because pain assessment relies largely on subjective self‐report and objective biomarkers remain limited, ML‐based approaches could contribute to improving risk stratification, diagnosis, prognosis, monitoring, and treatment planning. Fillingim et al. used ML methods with UK Biobank data to identify candidate pain biomarkers and emphasized the importance of integrating biological and psychosocial factors in pain prediction [69]. Similarly, Mackey et al. proposed that incorporating biomarkers into clinical decision‐support tools could improve personalized care, diagnostic accuracy, prognosis, and treatment selection [124]. Although these studies were not specific to stroke, they provide a framework that may inform the development of predictive models for CPSP.

Evidence from stroke populations further supports this approach. Asseyer et al. demonstrated that QST performed during the acute poststroke phase can identify sensory abnormalities associated with subsequent development of CPSP, suggesting potential value for future predictive models that help identify individuals at increased risk and support early interventions [125]. These findings align with the growing need for personalized assessment and prognostic tools that improve risk stratification and support individualized rehabilitation strategies for CPSP [120].

Despite this promise, ML applications to CPSP remain in the early stages, and no CPSP‐specific predictive or diagnostic models have been validated for clinical use. Previous work by Klit et al., Singer et al., and Yuan et al. highlighted the importance of somatosensory abnormalities, lesion location, and alterations in central pain‐processing pathways in the development and pathophysiology of CPSP. Collectively, these findings provide a rationale for developing multidimensional predictive models that integrate clinical characteristics, sensory dysfunction, lesion features, neuroimaging findings, and psychosocial factors. Given CPSP’s complex, heterogeneous nature, such approaches may facilitate earlier identification of individuals at increased risk, improve risk stratification, and support more personalized assessment and management strategies. Future research must establish the predictive performance, external validity, and clinical utility of these models before their implementation in routine clinical practice.

##### 3.4.10.2. Robotic Rehabilitation

Robotic‐assisted rehabilitation is increasingly used in stroke recovery and has shown benefits for motor function, mobility, and task‐oriented performance [126–140]. These systems also provide objective performance metrics, real‐time monitoring, and support intensive, adaptive rehabilitation protocols tailored to individual patient needs [129–131].

However, the clinical relevance of robotic‐assisted rehabilitation for CPSP remains uncertain. Most studies have focused on motor recovery and functional outcomes rather than pain‐specific measures, leaving insufficient direct evidence to establish its effectiveness in managing CPSP. Although some studies have reported beneficial effects on poststroke musculoskeletal pain conditions, such as hemiplegic shoulder pain [141, 142], these findings cannot be directly extrapolated to central neuropathic pain. Furthermore, evidence suggests that improvements observed with robotic rehabilitation often do not reach thresholds considered clinically meaningful (minimal clinically important difference, MCID), despite statistical significance [143, 144]. Improvements in motor or sensory function should therefore not be considered equivalent to analgesic benefits, particularly given the distinct central neuropathic mechanisms underlying CPSP. Therefore, while robotic rehabilitation remains a valuable component of stroke neurorehabilitation, its specific effectiveness for CPSP has not been established. Future studies should prioritize pain‐related outcomes to determine whether these technologies can meaningfully influence CPSP symptoms and associated disability.

ML and robotic rehabilitation both present promising opportunities to advance CPSP research and clinical care. However, implementing these technologies in clinical practice requires careful evaluation of the evidence and a clear distinction between established applications and those that remain exploratory. This approach supports responsible, evidence‐based integration of these technologies into neurorehabilitation practice. CPSP is challenging to diagnose due to its heterogeneity and variable treatment responses. Implementing a structured clinical approach can support earlier identification and more effective therapeutic decision‐making. Ultimately, integrating emerging technologies into a structured, evidence‐based clinical framework may support earlier identification, more precise assessment, and individualized management of CPSP, provided rigorous validation establishes their clinical utility.

#### 3.4.11. Clinical Approach to the Diagnosis of CPSP

Diagnosing CPSP is a clinical challenge because there is no clear gold standard test, and its symptoms often overlap with other types of PSP [10, 54, 58, 65]. Using a structured clinical approach can help clinicians recognize CPSP more reliably and make more accurate diagnoses. The proposed clinical diagnostic framework is summarized in Figure 1.

**FIGURE 1:**
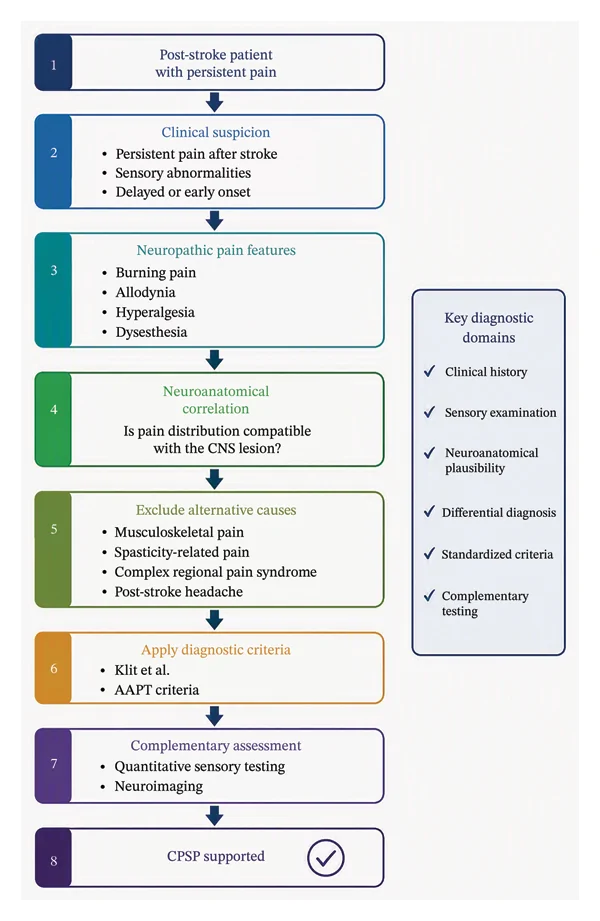
Clinical diagnostic framework for central post‐stroke pain (CPSP).

CPSP is often missed in clinical practice because its symptoms vary widely, and it frequently occurs alongside other types of poststroke‐related pain. The following step‐by‐step process is recommended to help guide clinical assessment and diagnosis:

1. Clinical suspicion: Consider CPSP in individuals who develop persistent pain following a stroke, particularly when pain is accompanied by sensory abnormalities [8, 10, 65].
2. Pain characterization: Assess the quality, intensity, temporal features, and distribution of pain, with particular attention to neuropathic characteristics such as burning pain, allodynia, hyperalgesia, and dysesthesia [10, 82, 84, 85].
3. Neuroanatomical correlation: Determine whether the distribution of pain and associated sensory abnormalities is anatomically consistent with CNS lesions [10, 13, 44].
4. Exclusion of alternative causes: Evaluate for other potential sources of PSP, including musculoskeletal pain, pain associated with spasticity, and complex regional pain syndrome. The coexistence of another pain condition does not necessarily exclude CPSP [8, 67, 86].
5. Application of neuropathic pain diagnostic criteria: Use established diagnostic frameworks and validated criteria, such as those proposed by Klit et al. or the ACTTION–AAPT, to strengthen diagnostic confidence [10, 58, 62].
6. Complementary assessment: When clinically indicated and available, incorporate findings from QST, neuroimaging, or other relevant assessments to further characterize somatosensory dysfunction and support the clinical diagnosis [12, 44, 125].

This systematic approach facilitates earlier identification of CPSP, reduces missed diagnoses, and strengthens neurorehabilitation decision‐making [10, 54, 65, 86]. Employing a structured diagnostic framework also aids in distinguishing CPSP from other PSP syndromes and guiding optimal management strategies. Ultimately, a structured clinical framework enhances CPSP recognition and supports more informed, individualized therapeutic decisions for this patient population.

#### 3.4.12. Therapeutic Hierarchy for the Management of CPSP

Managing CPSP is challenging because its underlying mechanisms are heterogeneous and individual responses to treatment vary [6, 10, 82]. Systematic reviews indicate that no single pharmacological or nonpharmacological intervention is effective for all patients, and that the limited effectiveness of isolated treatments supports an integrated, mechanism‐based, multimodal strategy [7, 82]. General neuropathic pain guidelines, such as the ICCPN, recommend tricyclic antidepressants, SSRIs, pregabalin, and gabapentin as first‐line options, with topical therapies and tramadol as second‐line, and strong opioids and botulinum toxin as third‐line [86, 99]. The hierarchy proposed below refines this general framework using CPSP‐specific evidence where available; where such evidence is limited, it retains the broader guideline recommendation. Nevertheless, randomized evidence specifically in CPSP remains scarce and heterogeneous, and a systematic review of clinical trials concluded that the available results were inconsistent with the main clinical practice guidelines [110]. The proposed therapeutic hierarchy for CPSP management is summarized in Figure 2.

**FIGURE 2:**
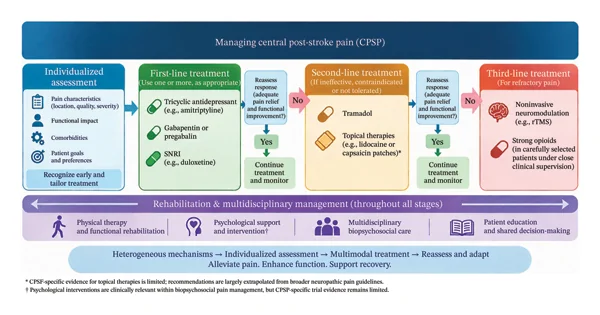
Therapeutic hierarchy for the management of central post‐stroke pain (CPSP).

The proposed hierarchy reflects not only the available evidence but also practical clinical considerations, including treatment accessibility, feasibility of implementation, tolerability, cost, and availability within routine rehabilitation settings.

##### 3.4.12.1. First‐Line Treatment

• Tricyclic antidepressants (e.g., amitriptyline).
• Anticonvulsants (e.g., gabapentin, pregabalin).
• Serotonin‐norepinephrine reuptake inhibitor (SNRI; duloxetine).

Tricyclic antidepressants and the gabapentinoids gabapentin and pregabalin are among the most consistently recommended pharmacological options for neuropathic pain, including CPSP [82, 86, 99, 145–147]. In the landmark placebo‐controlled trial of amitriptyline in CPSP, the drug produced a statistically significant reduction in pain compared with placebo [148], and a network meta‐analysis of antidepressants and anticonvulsants in CPSP identified pregabalin and gabapentin as among the most effective agents [149]. Duloxetine is included here at the same level as amitriptyline because, in the most recent systematic review and meta‐analysis specific to CPSP (42 studies, 1451 participants), duloxetine, amitriptyline, and rTMS were identified as the interventions with the most robust evidence of efficacy in this condition [7], a finding supported by a dedicated randomized, double‐blind, placebo‐controlled trial in which duloxetine significantly reduced pain intensity and improved disability in patients with moderate‐to‐severe CPSP [150]. SSRIs, included among first‐line options in general neuropathic pain guidelines such as ICCPN [99], are not emphasized here as a distinct first‐line option, since CPSP‐specific trial evidence for this drug class remains limited. Individual response and tolerability to first‐line agents vary considerably, and many patients achieve only partial relief [6, 82].

##### 3.4.12.2. Second‐Line Treatment

• Tramadol
• Topical therapies (e.g., lidocaine or capsaicin patches).

When first‐line treatments are ineffective, contraindicated, or poorly tolerated, clinicians may consider second‐line options. Neuropathic pain guidelines recommend tramadol and topical lidocaine or capsaicin patches as second‐line options [86, 99, 145, 146], consistent with the ICCPN framework. However, topical agents have been evaluated mainly in peripheral neuropathic pain. Unlike strong opioids, tramadol has a dual mechanism of action, combining weak μ‐opioid receptor agonism with serotonin and norepinephrine reuptake inhibition. Together with its more favorable safety profile, this supports its classification as a distinct second‐line therapy rather than within the strong‐opioid category [145, 146, 151, 152].

##### 3.4.12.3. Third‐Line Treatment

• Noninvasive neuromodulation techniques (repetitive transcranial magnetic stimulation, rTMS).
• Strong opioids, in carefully selected patients and under close clinical supervision.

For patients who remain refractory to pharmacological treatment, noninvasive neuromodulation and strong opioids constitute third‐line options. Although rTMS has demonstrated promising analgesic effects in CPSP, its placement as a third‐line intervention reflects not only the available evidence but also practical considerations such as limited accessibility, variability in stimulation protocols, and the need for specialized equipment and clinical expertise. rTMS has shown promising analgesic effects in CPSP: a meta‐analysis by Gurdiel‐Álvarez et al. reported a large effect size (SMD = −1.45, 95% CI −1.87 to −1.03) despite low overall quality of evidence [115], while an independent meta‐analysis by Liu et al. reported a similarly significant reduction (SMD = −1.15, 95% CI −1.69 to −0.61) across six RCTs [153]; stimulation parameters remain nonstandardized across studies [72, 111–113]. Repeated rTMS sessions may provide clinically meaningful and, in some patients, prolonged pain relief [114], and in the systematic review by Tamasauskas et al., rTMS was among the interventions with the most robust evidence for CPSP, within a neuromodulation category showing a moderate pooled effect (SMD = −0.64) [7]. Strong opioids should be reserved for carefully selected patients and used under close clinical supervision, since evidence for opioid efficacy in central neuropathic pain overall is limited and the analgesic response appears smaller than in peripheral neuropathic pain [154–156]. Botulinum toxin, listed as a third‐line option in general neuropathic pain guidelines [99], is not included here as a CPSP‐specific recommendation, as no trials evaluating its use in this condition were identified.

##### 3.4.12.4. Rehabilitation and Multidisciplinary Management

• Physical therapy and functional rehabilitation.
• Psychological interventions.
• Multidisciplinary management.

Nonpharmacological and supportive interventions should complement pharmacological treatment. Physical therapy and functional rehabilitation support recovery and may contribute to pain control, showing a nonsignificant but directionally favorable pooled effect in the most recent systematic review [7, 82, 107, 108], while multidisciplinary, biopsychosocial management addresses the emotional, cognitive, and functional dimensions of chronic pain [157]. Notably, the most recent systematic review identified no studies of psychological interventions specifically in CPSP, underscoring a distinct gap that warrants dedicated to future research [7].

CPSP management should therefore integrate pharmacological and nonpharmacological strategies, tailoring treatment to each patient’s symptoms, functional limitations, comorbidities, treatment response, and personal goals [82, 111]. Early recognition and timely intervention are essential, since adequate CPSP treatment not only alleviates pain but also supports rehabilitation and functional recovery [65, 108].

## 4. Conclusion

CPSP continues to be a clinically challenging and incompletely understood condition, marked by significant heterogeneity in its underlying mechanism and clinical presentation. Instead of stemming from a single pathophysiological process, CPSP results from complex interactions among sensory, neuroplastic, inflammatory, and psychosocial factors. This complexity leads to diagnostic uncertainty and explains why current treatments often have inconsistent results.

From a clinical perspective, the persistent underdiagnosis of CPSP highlights significant limitations in conventional assessment frameworks, which frequently fail to capture its neuropathic and multidimensional characteristics. The absence of structured, condition‐specific diagnostic tools further contributes to delayed identification and timely management.

Likewise, the limited effectiveness of pharmacological interventions indicates that CPSP cannot be adequately managed with isolated treatment modalities. Instead, optimal care requires integrated, mechanism‐based strategies that address its neurobiological, psychological, and functional dimensions.

Although emerging technologies like ML and robotic rehabilitation demonstrate substantial potential in poststroke recovery, their application to CPSP remains nascent. Current evidence underscores key limitations, such as the absence of CPSP‐specific predictive models and a lack of clinically meaningful improvements in pain‐related outcomes. In this context, it is fundamental to avoid overestimating the potential of these technologies. While promising, their clinical adoption necessitates further validation and methodological refinement.

Collectively, these findings highlight the need to move beyond a descriptive and fragmented understanding of CPSP toward an integrated, patient‐centered care model. This approach should include precise diagnostic frameworks, personalized therapeutic hierarchies, and the judicious integration of emerging technologies. Ultimately, progress in CPSP management will require not only technological advances but also stronger conceptual integration across disciplines, with an emphasis on clinically meaningful outcomes for patients.

## Author Contributions

Cristhian Mendoza and Carolina Carmona: conceptualization, investigation, methodology, analysis and interpretation, writing–original draft, and writing–review and editing.

## Funding

No funding was received for this research.

## Disclosure

All authors read and approved the final manuscript.

## Ethics Statement

Approval was waived.

## Consent

The authors have nothing to report.

## Conflicts of Interest

The authors declare no conflicts of interest.

## Data Availability

Data sharing is not applicable to this article as no datasets were generated or analyzed during the current study.
